# “It’s MAGIC” - development of a manageable geriatric assessment for general practice use

**DOI:** 10.1186/s12875-014-0215-4

**Published:** 2015-01-22

**Authors:** Tanja Barkhausen, Ulrike Junius-Walker, Eva Hummers-Pradier, Christiane A Mueller, Gudrun Theile

**Affiliations:** Institute for General Practice, Hannover Medical School, Carl-Neuberg-Strasse 1, 30625 Hannover, Germany; Department of General Practice, University Medical Centre Goettingen, Humboldtallee 38, 37073 Goettingen, Germany; Santémed Health Center, Seebahnstrasse 89, 8036 Zuerich-Wiedikon, Switzerland

**Keywords:** Geriatric assessment, General practice, Health services for the aged, Questionnaire design, Chronic diseases

## Abstract

**Background:**

Geriatric assessments are established tools in institutional care since they enable standardized detection of relevant age-related disorders. Geriatric assessments could also be helpful in general practice. However, they are infrequently used in this setting, mainly due to their lengthy administration. The aim of the study was the development of a “manageable geriatric assessment – MAGIC”, specially tailored to the requirements of daily primary care.

**Methods:**

MAGIC was developed based on the comprehensive Standardized Assessment for Elderly People in Primary Care (STEP), using four different methodological approaches: We relied on A) the results of the PRISCUS study by assessing the prevalence of health problems uncovered by STEP, the importance of the respective problems rated by patients and general practitioners, as well as the treatment procedures initiated subsequently to the assessment. Moreover, we included findings of B) a literature analysis C) a review of the STEP assessment by experienced general practitioners and D) focus groups with general practitioners.

**Results:**

The newly created MAGIC assessment consists of 9 items and covers typical geriatric health problems and syndromes: function, falls, incontinence, cognitive impairment, impaired ears and eyes, vaccine coverage, emotional instability and isolation.

**Conclusions:**

MAGIC promises to be a helpful screening instrument in primary care consultations involving elderly multimorbid patients. Applicable within a minimum of time it still covers health problems highly relevant with regard to a potential loss of autonomy. Feasibility will be tested in the context of a large, still ongoing randomized controlled trial on “reduction of potentially inadequate medication in elderly patients” (RIME study; DRKS-ID: DRKS00003610) in general practice.

## Background

The aging population is a well-known challenge for most European health care systems. General practitioners (GPs) have a key role in health care for elderly people; this applies also to Germany. Consequently German GPs will have to deal increasingly with typical conditions and health problems of old age, especially with multimorbidity.

Geriatricians, who are routinely concerned with the complexity of health problems in old age, established the multidimensional and structured approach of comprehensive geriatric assessments (CGAs) to uncover relevant functional, cognitive, emotional and social disorders. CGAs enable doctors to monitor and evaluate the health conditions of elderly patients over an extended time period and facilitate sustainable therapeutic decisions. Yet, most geriatric assessments are tailored to the specific needs of institutional care populations and focus on function and cognition, but rarely suit primary care providers and older people living in the community. Therefore, a collaboration of European GPs developed the STEP in the 1990s [1]. STEP is an evidence-based CGA [2], which contains 44 items. It is mainly a questionnaire, complemented by some laboratory values and performance tests. Although STEP has been successfully used in several studies in Austria [3], Italy [4] and Germany [5,6], it has never been applied routinely in primary care settings, probably due to its high time requirements. Nevertheless in evaluation studies subsequent to our PRISCUS study [6], GPs rated STEP as very helpful to uncover so far unknown and unreported health problems in older patients.

Hence we aimed to create a short “manageable geriatric assessment” (“MAGIC”) – a geriatric assessment tailored to the specific requirements of tightly scheduled daily primary healthcare on the basis of STEP, comprising topics considered important by GPs and patients. The need for such an approach has recently been underlined by the findings of Min et al.. They demonstrated that in general practice, patients with geriatric morbidities often receive poorer quality of care than those with non-geriatric medical conditions. They suggest more systematic approaches to overcome this flaw and to improve outcomes [7]. Different geriatric assessments have already been developed and performed in primary care like the brief assessment questionnaire in context of the MRC Trial, which was probed in 33000 patients [8], the Easy Care Assessment [9], the Easy Care TOS [10] to identify frail people or SPICE a short instrument to detect “unmet needs” in older people in the community [11].

MAGIC is intended as a very brief screening tool for the heterogeneous population of older patients consulting in general practice and may help to uncover so far unidentified and unmentioned health problems. We define health problems as issues having the potential to harm the health status of a patient. MAGIC is not intended to be used specifically in elderly patients with high risk of frailty or functional decline, but rather for a larger screening. In case MAGIC detects relevant health problems, more specified assessment tools and diagnostic tests should be applied. Predefined diagnostic or therapeutic options cannot be given from our side - as this was not part of our project.

MAGIC was developed in the context of a large randomized clinical trial (RCT) on medication in the elderly (RIME). The aim of this article is to present the methodology and results of our studies to develop the feasible MAGIC assessment based on the comprehensive STEP assessment.

## Methods

The MAGIC development was composed of two parts: a pre-selection part and a final selection part.

The pre-selection part consisted of four different methodological approaches of information gathering to identify the most relevant items of the STEP assessment and possibly additional items:

A: Data on prevalence, importance ratings of GPs and patients participating in the preceding PRISCUS study [6] and treatment initiated afterwards of each of the 44 STEP items (see Table 1).
B: Literature search focused on contents of other health care assessments for senior citizens living in the community
C: Review of the STEP assessment by experienced GPs
D: Focus group discussions with GPs

**Table 1:** **Overview of STEP Items, results of the pre-selections, instruments, and qualitative arguments given in pre-selections A and D**

**STEP item** ***(plus instrument if included in MAGIC)***	**Selected in pre-selection part**				**Qualitative arguments given**	
	**A**	**B**	**C**	**D**	**in pre-selection part A**	**in pre-selection part D**
**Decreased performance with everyday tasks** *COOP/WONCA Charts* [12]	x		x		A question concerning function was included because ADL is a crucial content of a geriatric assessment	
Decreased maximal exercise capacity for two minutes						
Problems with BADL		x		x		Important for independence
Problems with IADL		x	x	x		
Problems with housing						
**No help in emergency** *Lachs* [13]			x	x		A social network is considered important, especially as a missing network leads to insecurity and anxiety
No help in sickness						
**No person to trust** *OARS* [14]		x		x		
Being a caregiver	x		x		Being a caregiver is highly important for the patient with regard to preserving autonomy	
Financial problems						
Breathlessness	x				Health problem was rated as important for GP and patient	
Chest pain	x				Chest pain is often new to GP, important for the patient; interventions are often planned	
Claudication	x				Claudication is often new to GP; health problem is important for doctor and patient; in many cases interventions were planned and conducted	
Dizziness	x				Interventions are often planned and conducted subsequently to the assessment	
Difficulty sleeping	x				Sleeplessness has high prevalence and is often new to the GP	
Pain	x		x		Pain was included because of its high prevalence	
Weight loss	x				Interventions are often planned and conducted subsequently to the assessment	
**History of falls** *Tinetti* [15]	x	x	x	x	Falls are often new to GP	All GPs agreed that this item is essential in a geriatric assessment
History of cardiac infarction						
History of stroke						
History of fractures						
Problems with chewing			x			
**Urinary incontinence** *Sandvick* et al. [16]	x	x	x		Urinary incontinence has a high prevalence and is often new to the doctor, patients often keep quiet about the problem because of shame	
Fecal incontinence/constipation	x	x			Problem is important for the patient	
**Problems with vision** *Lambeth Disability Questionnaire* [17,18]	x	x	x	x	There was consensus among the researchers that this health problem must be included as “basic GA item”	Despite some controversies (no consequence, most patients regularly visit an ophthalmologist), most GPs recommend the inclusion of this item because patients do not address the problem on their own
**Problems with hearing** *Lambeth Disability Questionnaire* [17,18]	x	x	x	x	There was consensus among the researchers that this health problem must be included as “basic GA item”	Despite some controversies (can easily be detected without an assessment), most GPs recommend the inclusion of this item because patients do not address the problem on their own. Furthermore, good hearing is important for social activities.
**Depression** *Whooley* et al. [19]	x	x	x	x	Health problem is important for GP and patient; many planned and conducted interventions	All GPs agreed that this item is essential in a geriatric assessment
					There was consensus among the researchers that this health problem must be included as “basic GA item”	
Mourning						
Loneliness						
Anxiety	x				Anxiety is important for the patient; monitoring is important for differentiation between depression and anxiety	
Smoking						
Alcohol abuse						
No healthy diet		x		x		Especially the ability to prepare a meal seems to be important for a balanced diet, the item is closely linked to the IADL aspects
Too little exercise						
**Immunization missing or unknown** *STEP* [1]	x				Very high prevalence, problem is often new to GP	
Problems with medication		x	x	x		The item seems to be very important for GPs because lack of compliance, use of OTC drugs, and prescriptions of other medical specialists unknown to the GP are frequent problems
Hypertension						
Arrhythmia						
High blood sugar or known diabetes						
High cholesterol						
Thyroid dysfunction						
**Abnormal clock drawing test** *Clock drawing test* [20,21]	x	x	x	x	Problem is often new to GP; A basic cognition test is central to a geriatric assessment	All GPs agreed that this item is essential in a geriatric assessment
Abnormal timed-up-and-go			x			All GPs agreed that this item is essential in a geriatric assessment
Foot problems	x		x		High prevalence	

Items selected for MAGIC are in bold text type.

For final selection, the collected quantitative and qualitative results of the pre-selection were scrutinized by a team of researchers and GPs and merged into the ultimate MAGIC instrument. This can be considered as a qualitative content validity analysis as introduced by Lawshe [22].

Feasibility is tested in the context of a currently ongoing large controlled randomized study (RIME) and is not the subject of this paper.

### Pre-selection

- Pre-selection part A: Analysis of PRISCUS results
  In the PRISCUS study [6], conducted from July 2008 to May 2010, patients of 72 years and older received the STEP assessment in their GP practice. On the basis of the individual STEP results, 396 patients rated the importance of each of their individual health problems (a). The respective 46 GPs independently evaluated each of their patients’ problems – initially according to its relevance for care (b), whether the problem was new to them (c) and whether an intervention was planned (d). At the end of the study period GPs also had to indicate whether the planned intervention had been carried out (e). In a first analysing step, four scientists independently evaluated the ratings a – e by patients and doctors, and additionally the prevalence of each item (f), and formed an individual opinion about recommendation of every single item for enclosure in MAGIC. The subjectivity of this approach was intended to preserve a broad coverage of scientific appraisal concerning the different aspects of ratings carried out by the study population.
  The selections were subsequently compared: if more than two of the four scientists valued a health problem as relevant to be enclosed into a short assessment, the item was selected as potentially eligible for MAGIC. Conversely, if more than two researchers assessed a health problem as unimportant, the item was excluded from the selection. If an item was equally valued, pros and cons were discussed until a consensus on in- or exclusion was achieved.
- Pre-selection part B: Literature search
  We performed a PubMed literature search and review to understand which health problems had previously been considered important for the assessment of older patients’ health in an international context and in the eyes of other research groups. For that purpose, we conducted an online search with all possible combinations of the following terms:
  “geriatric assessment ***AND*** short *OR* comprehensive ***AND*** primary care *OR* general practice *OR* outpatient”.
  Five hundred and two publications were screened for eligibility. Additionally, we consulted grey literature (via the internet), and further assessments were searched by hand. Finally, 19 assessment instruments were identified as suitable for our investigation. All health problems extracted from this literature were ranked according to frequency of occurrence. Finally we determined the average of occurrence in the ranking list, which was 6.5 times. If an item occurred more than this average (7 times or more) it was included into the result list of study part B – independently of its consideration in STEP.
  **List of assessments considered in literature analysis**
  CANE Assessment [23]
  SPICE Assessment [11]
  Bremer Vorsorgeuntersuchung (Bremen Check-Up) [24]
  MRC Trial [8]
  Nottingham Health Profile [25]
  DUKE assessment [26]
  Sickness Impact Profile [27]
  Lachs [13]
  Assessment published by Davidson et al. [28]
  Assessment published by Caplan et al. [29]
  Assessment published by McCusker et al. [30]
  EASY Assessment [31]
  AGAST Assessment [32]
  Geriatric Assessment/University of Bern [33]
  EBM2000_Geriatrisches Basisassessment [34] (Basic Geriatric Assessment)
  Assessment published by Fleming et al. [35]
  Guidelines & Protocols Advisory Committee (GPAC). Frailty in Older Adults- Early Identification and Management [36]
  New Zealand screening and proactive assessment [37]
  GP Guideline: Hausärztliche Leitlinie: Allgemeine Geriatrie Hessen [38]
- Pre-selection part C: Review of STEP by individual GP
  A sample of fifteen GPs, associated teachers of the Institute of General Practice in Hannover, were given a questionnaire listing the 44 items of STEP from which they were asked to select a maximum of 20 particularly important items in a simple checkbox system. These GPs were not informed about the ratings and results of pre-selection part A and B. A free text field was offered for comments. Seven forms were sent back anonymously. In the following analysis, we ranked the selected STEP items according to their frequency of occurrence. All items chosen more than five times were added to the list of items to be evaluated in the final selection part.
- Pre-selection part D: Focus groups with GPs
  To get a deeper insight into opinions and interests of practising primary care physicians, we conducted three focus groups. During the focus groups, the participants (five to eight GPs per focus group; 20 in total) were initially asked for their wishes and needs concerning a GA suitable for primary care in general. The time aspect (tolerated length of administration in day-to-day practice) was addressed in particular. Afterwards, GPs discussed which health problems should be included into a short geriatric assessment. The resulting list was compared openly with the temporary findings of the MAGIC development process existing at that point of time. Focus group participants discussed and confirmed or rearranged the temporary classification and decided on ambiguous items. The discussions were recorded and afterwards analysed by mind mapping [39]. Additionally, results were visualised using a freeware program (FreeMind, Softonic) for final analysis. All participants who attended the focus groups agreed to the recording and data storage.

### Final selection

Five scientists, three of whom were also GPs, took part in a meeting in which all items considered relevant in at least three of the four pre-selection parts A to D were again closely reviewed and intensively discussed. In this conference, the most important items were finally selected for the MAGIC assessment.

### Ethics

The development of the MAGIC assessment was performed as a part of the RIME study. The RIME project was approved by the ethics committee of Hannover Medical School (project-ID: 1361–2012).

## Results

### Results of the pre-selection parts A-D

- Pre-selection part A: Analysis of PRISCUS results
  We included 19 health problems in our pre-selection with regard to the above mentioned criteria a–f (Table 1).
- Pre-selection part B: Literature search
  By ranking the health problems that are mentioned in internationally available GA with regard to their frequency of occurrence in selected articles, we identified a set of 12 health problems that are very frequently addressed in GA. The selected items are listed in Table 1. All items were already part of the STEP. Our literature search did not reveal additional health problems to be considered.
- Pre-selection part C: Review of STEP by individual GP
  We identified 15 health problems that were important for most of the responding GPs. We received only a few annotations concerning the “top 15 items” (Table 1), but instead plenty of comments with regard to items that were considered inadequate for the assessment by the GP. For example, one GP argued that all items that were obtained elsewhere (and probably more reliably) should be excluded from a GA, such as laboratory findings or pre-existing chronic conditions.
- Pre-selection part D: Focus groups with GPs
  Most of the participating GPs expressed that in general, geriatric assessments might be useful instruments. But due to time pressure in daily routine care they are not used. A short geriatric assessment adapted to the requirements of a primary care practice should not take longer than ten to fifteen minutes. Twelve health problems were considered particularly relevant by the participants. These health problems and also comments concerning these items are shown in Table 1.

### Results of the final selection part

**Figure 1:**
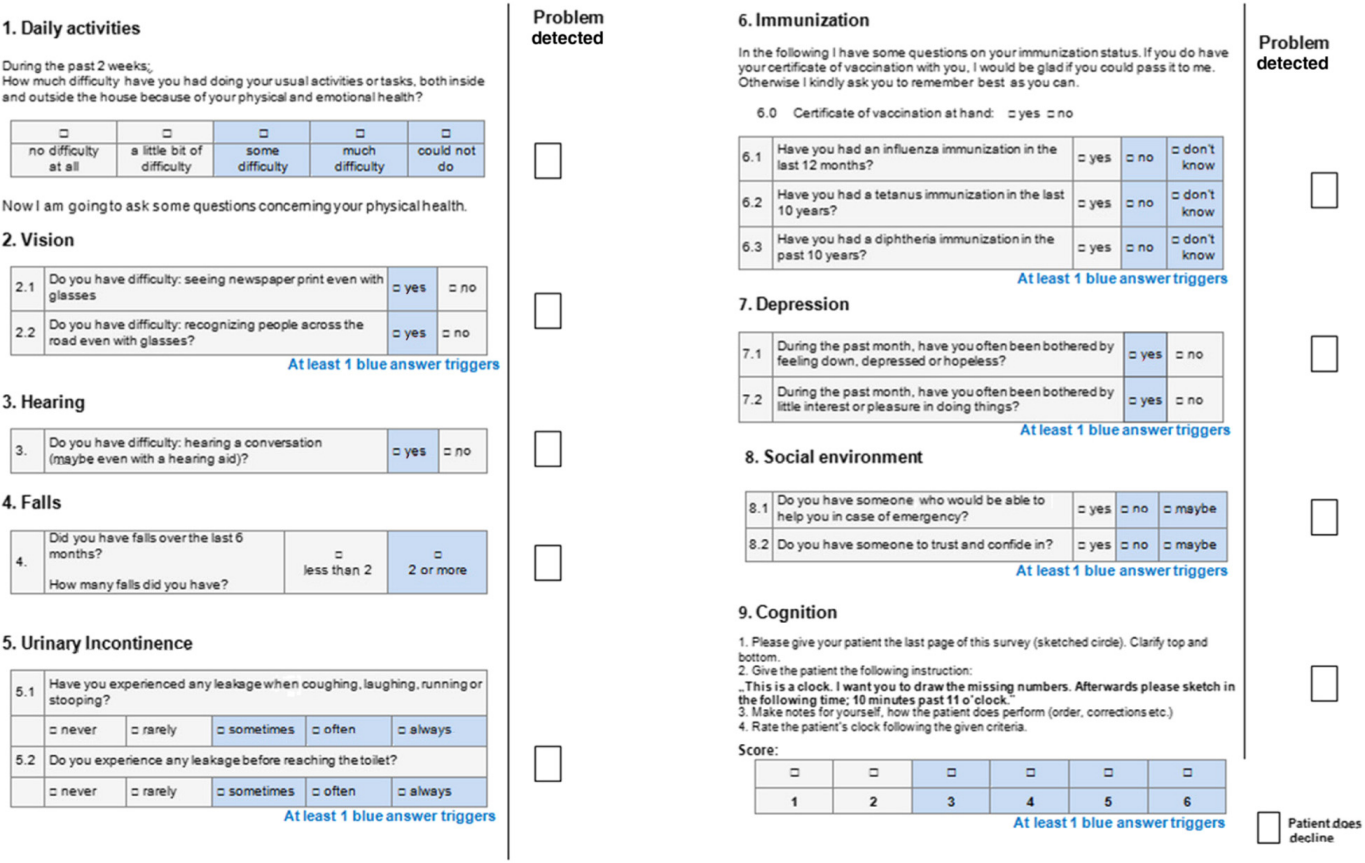
**MAGIC assessment (abridged).** Shows an abridgement of the MAGIC assessment. The analysis is directly integrated and explained in the assessment and trigger fields are highlighted in blue. For a better overview, detected problems may also be highlighted in the sidebar.

Items that were considered important in at least three of the four pre-selection parts were critically discussed in a round of five scientists and GPs with regard to their eligibility. The item “immunization missing or unknown” represents an exception, as it was solely considered because of its high prevalence rate in PRISCUS study and the high number of interventions that followed. Finally, nine topics were selected for the MAGIC assessment (Table 1, Figure 1).

## Discussion

In this study, we designed a short ‘basic geriatric assessment’ purpose-built for routine application in general practice.

Initial data collected in the RIME study revealed that it will take approximately 15 minutes to implement MAGIC. This is in strong contrast to comprehensive geriatric assessments that regularly require a time investment of 45 minutes or more. MAGIC is easy to understand and evaluate, because the analysis tool is included in the questionnaire.

As we developed MAGIC in the context of a large trial on medication in the elderly, questions with regard to medications were not additionally included although this topic was of relevance for GPs in the review of the STEP and in the focus groups. The performance of MAGIC in the context of a routine visit should be completed by a medication review, considering polypharmacy and potentially inadequate medication (e.g. using the German PRISCUS list [40]).

In the following, we will give a short overview of the existing evidence on health problems we finally included in MAGIC.

### STEP health problems selected for MAGIC

- Functional impairment
  Monitoring of function is a basic tool in most comprehensive geriatric assessments, since functional impairment is a common problem of older patients and has strong influence on morbidity and mortality [41]. Moreover, functional decline is found to be correlated with decreases in lifestyle as well as quality of life [42].
  However, in our final decision round the opinions regarding this item were controversial. Despite the fact that in the development process most of the participating GPs valued the item “function” as very important, some of the participants of the final decision round cautioned against a low acceptance by GPs for an assessment containing questions concerning function (due to the fact that functional problems are often known by the GP). We found an agreement by including a single basic question, derived from COOP/WONCA charts [12] rather than a more comprehensive evaluation of the topic “function”. The latter may follow if function is identified to be a point of concern.
- Impairment of vision and hearing
  Visual impairment and hearing loss are quite common in elderly patients [43,44]. However, both problems are often unrecognized and untreated, though they are correlated with a variety of adverse effects. For visual impairment, an increased mortality risk and additional decrease in quality of life as well as increases in falls, medication noncompliance, automobile accidents, and hip fractures have been demonstrated [45-50]. Likewise, hearing loss is linked to detrimental effects as it is known to be associated with social isolation, functional decline, decreased quality of life, depressive symptoms, and cognitive deficits [51].
  Since it has been widely demonstrated that early detection of sensory problems are helpful to protect from harm [52] and the regular screening for hearing loss has been previously recommended by several institutions [51], we decided to include the evaluation of vision and hearing in our assessment.
- Falls
  Falls have an incidence of about 30% per year in the population age group of 65 and over [53]. They are caused by a variety of both, intrinsic and extrinsic factors. The incidence for falls increases with age [54] with a higher prevalence for women. About 5–6% of the accidental falls result in a fracture or other serious injuries [55] quite frequently leading to permanently decreased mobility and an increased mortality. Fall prevention is a highly important issue in the care of elderly patients [56].
- Urinary incontinence
  Urinary incontinence has a prevalence of 15–30% in the elderly population. Women are more often affected by urinary incontinence, with a female to male ratio of 2:1 [57]. Urinary incontinence is an important issue for elderly patients since it is associated with ADL-specific functional decline [58], frailty [59], reduced quality of life [60], depression, and social isolation [61].
- Immunization status
  For immunization, clear guidelines do exist and adherence to these should be fostered for the benefit of the individual and the public. Furthermore, the solution to the problem of an insufficient immunisation status is fast, cheap and easily done. We decided to include the monitoring of vaccination mainly for one reason: in the PRISCUS results, missing or unknown vaccinations had the highest prevalence of all STEP items.
- Depression
  Prevalence rates of clinically relevant depression in the elderly population are up to 36% [62]. However, symptoms for a depressive disorder are often masked by other symptoms in older patients, such as impaired cognition [63] psychomotor agitation or other symptoms. Older persons often express a multitude of problems due to age-associated changes, making the diagnosis of depression particularly difficult [63].
- Social background
  Due to demographic changes and decreasing family ties, social isolation can become more frequent. The number of older people living in single person households is high in Germany: 44% of women and 18% of men older than 65 years lived in one-person-households; in addition the percentages increase with further age [64]. Although in a recent German cohort study an influence of social relations on cognition or mortality could not be found [65], a systematic review indicated that strong social relationships increase the likelihood of survival by 50% in the 148 studies included [66].
- Cognitive impairment
  Within the public, as well as in the medical community, dementia is probably the best recognized geriatric syndrome. It is expected that 2% to 8% of the age group 65 and over and 30% of those aged 90 years or more suffer from dementia [67,68], and that the total number of people with dementia will considerably increase within the next 2 decades. GPs, as the main contact partners for most elderly people, are well trained to detect and treat dementia. Nevertheless many GPs know their patients for such a long period, that small change in their behaviour or a well concealed increasing forgetfulness may be overlooked for a certain time. It is evident a geriatric assessment must contain a short cognitive test. Since the well-established clock drawing test has already been used in the STEP assessment, it was considered suitable for the MAGIC Assessment. In the PRISCUS study population 31% of the patients had a conspicuous result. The clock drawing test first introduced by Shulman 1986 is largely independent of education and socio-economic status [20,21]. However, GPs should make themselves aware that further clinical explorations as well as neuropsychological testing, neuro-imaging and possibly an interview of relatives, is required to confirm a diagnosis of dementia.

### Limitations

We want to point out that our selection process has not been limited to health problems already being included in STEP. The literature analysis looked openly at different GA, which enclosed items that were not contained in STEP. However, all of these items were later deleted from further investigation, since our approach relied on the frequency of occurrence. It turned out only health problems already enclosed in STEP were frequently parts of other assessments.

Since we initially deliberatively chose a broad and qualitatively oriented approach in order to be able to consider different existing instruments and GPs expectations, a pure statistical procedure to comprise STEP (e.g. by item-to-total correlation and calculating Crombach’s α such as done by Overcash et al. [69]) was never intended.

There is only a small number of participating GPs in the pre-selection part C. The originally planned large Delphi-survey had to be discarded due to its high requirements of time and effort on side of the participants. Instead, experienced GPs subjectively prioritized the STEP items. The individual preferences and comments of the participants enriched knowledge, and gave us important stimuli for the focus group guidance and discussion itself.

We think that the combination of the four different approaches constitutes a substantial methodological triangulation, complementing and enhancing one another.

### Next steps

Feasibility tests and evaluation of MAGIC is carried out in the context of the RIME study. Results will be obtained by the end of 2015. Future studies should address different test criteria as validity, reliability, and also statistics concerning the predictive value with regard to single outcome parameters (as e.g. functional decline, quality of life).

## Conclusion

We aimed to create a manageable geriatric assessment, since we are convinced that GPs need feasible approaches to support elderly primary care patients. The basis of our study was the comprehensive STEP assessment. We chose four different approaches to extract the most relevant aspects of a geriatric assessment in primary care and made a fifth and final step in conducting a consensus meeting. Thus, we performed a methodological “triangulation” by gathering information in different analytical and qualitative approaches. Congruence of extracted items between different selection parts was high, which underlines the assumed significance of items finally chosen. In the course of development of this approach, GPs and researchers decided in favour of inclusion of typical geriatric syndromes: immobility, instability, incontinence, cognitive impairment, impaired ears, eyes, and immunity, emotional instability, and isolation. The newly created MAGIC assessment promises to be a helpful screening instrument in primary care consultations with elderly patients since it highlights relevant health problems within only 15 minutes of application. Furthermore, MAGIC may be delegated to a practice nurse or other assisting personal in general practice after minimal training. Due to the integrated analysis as well as its simple structure and problem reporting, assessment results are clearly presented in the form.

Finally, we propose that the use of MAGIC will give valuable information to the GP and will contribute to a high quality of medical care of older patients in general practice.

## Abbreviations

BADL: Basic activities of daily living
CGA: Comprehensive geriatric assessment
GA: Geriatric assessment(s)
GP: General practitioner(s)
IADL: Instrumental activities of daily living
MAGIC: Manageable geriatric assessment
OARS: Older Americans Resources and Services
RCT: Randomized clinical trial
RIME: Reduction of inappropriate medication of elderly patients in primary care
STEP: Standardized assessment of elderly people in primary care
STIKO: The German Standing Committee on Vaccination (Ständige Impfkommission)
